# Synthetic biology empowers development of traditional Chinese medicine

**DOI:** 10.1016/j.chmed.2025.11.010

**Published:** 2025-11-20

**Authors:** Chun Li

**Affiliations:** Department of Chemical Engineering, Tsinghua University Center for Synthetic and Systems Biology, Tsinghua University Key Laboratory for Industrial Biocatalysis of Ministry of Education Beijing Key Laboratory of Recombinant Protein Synthetic Biomanufacturing State Key Laboratory of Green Biomanufacturing Beijing 100084, China

Natural products are the most precious gifts bestowed by nature upon humanity, serving as vital guardians for sustaining human reproduction and safeguarding health. Through the struggle against natural disasters and diseases, humans have developed a unique tradition of herbal medicine. In China, the use of plants for medicinal purposes and health preservation dates back over two thousand years as traditional Chinese medicine (TCM). With their diverse molecular structures, natural products have become a crucial source for the development of active pharmaceutical ingredients.

However, the simplest and most direct way to obtain these natural products is through chemical engineering extraction using wild or cultivated plant sources. This method of acquisition, which visibly damages ecosystems and vegetation, is clearly unsustainable. Although chemists have developed total synthesis routes for numerous bioactive natural products, issues such as numerous synthetic steps, lengthy production processes, difficulties in selectivity control, and overall low yields severely constrain their industrial production and application.

In recent years, advancements in systems biology have facilitated the elucidation of natural product biosynthetic pathways and the identification of key enzymes, gradually breaking through the “black box” enigma of metabolite synthesis. Synthetic biology has driven the development of novel production modes for heterologous biosynthesis of natural products in microbial cell factories, enabling synthetic biologists to construct cell factories using homologous or heterologous cells for the total synthesis of natural products.

## 1 Synthetic biology of natural products from traditional Chinese medicine

Traditional Chinese medicine (TCM), with profound chemical complexity and centuries-old therapeutic wisdom, provides the material basis of its clinical efficacy. Yet, the sustainable utilization and modernization of TCM are confronted with pressing challenges, including the overexploitation of wild resources leading to biodiversity loss, low yields of bioactive natural products, and difficulties in standardization. Globally, we are in the era of the fourth industrial revolution, which was characterized by the pursuit of energy-saving, emission-reducing, and high-efficiency green manufacturing. In this context, synthetic biology has been widely recognized as a foundational theory and key enabling technology for achieving green manufacturing across sectors, including pharmaceuticals and food production, sparking intense international competition. Recently, synthetic biology has emerged as a transformative paradigm, integrating principles from biology, engineering, and informatics to address these limitations in TCM. It offers an innovative approach to design and construct the biological systems capable of producing valuable natural compounds in a sustainable, efficient, and controllable manner. Vigorously developing synthetic biology is crucial for seizing the strategic high ground in this burgeoning bio-economy. By focusing on the elucidation and reconstruction of biosynthetic pathways, synthetic biology enables the green production of natural products through microbial cell factories. Compared to conventional biotechnological approaches, synthetic biology demonstrates distinct advantages in terms of sustainability, precision, and scalability, serving as a driving force behind the ongoing bio-manufacturing revolution.

## 2 Elucidating biosynthetic pathways in medicinal plants

A key approach in synthetic biology is to rebuild the biosynthetic pathways of natural products in microorganisms, such as *Saccharomyces cerevisiae* or *Escherichia coli*. To do this effectively, researchers need a thorough understanding of the main enzymes that drive the biosynthetic process, as well as the complex regulatory systems that control when and how these genes are expressed. Recent advances in multi-omics technologies — including genomics, transcriptomics, proteomics, metabolomics, and spatiotemporal omics — have facilitated the discovery of previously unknown enzymes involved in natural product biosynthesis. Following gene discovery, functional characterization of these enzymes in model microbial hosts is essential to validate their catalytic activities, substrate specificities, and regulatory roles. Expressing plant biosynthetic genes in well-characterized microbial systems allows for precise control over pathway components within a simplified and genetically tractable environment, thereby facilitating the identification of rate-limiting steps, key intermediates, and regulatory nodes. Furthermore, the integration of systems biology approaches — encompassing computational modeling, experimental validation, and network analysis — provides deeper insights into the complex regulation of natural product biosynthesis. Techniques such as co-expression analysis, gene cluster identification, metabolite profiling, deep learning-based pathway inference, and protein-protein interaction studies collectively contribute to the construction of dynamic, predictive models of metabolic networks. These models serve as invaluable guides for designing targeted metabolic engineering strategies, including metabolic flux optimization, yield enhancement, and pathway balancing.

## 3 Reconstructing natural product biosynthetic pathways

Synthetic biology has proven to be a powerful platform for the metabolic engineering of plant-derived natural products. Over the past decade, significant progress has been made toward the microbial production of several high-value TCM compounds, bringing them closer to commercial availability. By introducing synthetic or semi-synthetic biosynthetic pathways into microbial or even plant-based hosts, researchers have successfully engineered the production of complex molecules such as paclitaxel precursors, vinblastine, noscapine, and artemisinin. Among these achievements, the engineering of the artemisinin biosynthetic pathway in yeast stands as a landmark success, demonstrating the feasibility of producing structurally complex, plant-derived therapeutics through microbial fermentation. Likewise, microbial cell factories — particularly engineered yeast strains — have been utilized to produce pharmaceutically relevant compounds such as glabridin and glycyrrhizic acid derivatives, achieving production efficiencies that surpass those of traditional extraction methods by orders of magnitude.

Nevertheless, translating these laboratory successes into cost-effective, scalable industrial processes remains a formidable challenge. One major bottleneck lies in the incomplete elucidation of biosynthetic pathways for many TCM-derived compounds, particularly those that require extensive tailoring steps such as glycosylation, acylation, isopentenylation or methylation. Moreover, many plant-derived enzymes — especially cytochrome P450 monooxygenases, which often depend on specific redox partners — exhibit low catalytic activity, poor stability, and limited compatibility within the microbial hosts. Additional challenges include the potential toxicity of certain intermediates or end products to microbial cells, as well as metabolic imbalances that may result in the accumulation of toxic byproducts or the diversion of carbon flux toward competing endogenous pathways. Addressing these limitations will require integrated strategies spanning enzyme engineering, pathway optimization, and host cell engineering.

## 4 Innovative strategies for enhancing microbial production systems

To overcome current limitations and push the boundaries of microbial biosynthesis, innovative strategies at the interface of synthetic biology, systems biology, and artificial intelligence (AI) are being actively explored. AI and machine learning tools are increasingly employed to predict the functions of uncharacterized enzymes, uncover novel biosynthetic routes, and guide pathway design. These computational approaches, when integrated with high-throughput experimental data, enable a more rational and efficient exploration of chemical and enzymatic space. At the molecular level, protein engineering through rational design and directed evolution plays a critical role in enhancing the catalytic performance, stability, and substrate specificity of key enzymes. Complementing this, modular and dynamic pathway regulation strategies at the systems level allow for the independent optimization of individual pathway modules and the implementation of real-time feedback control to fine-tune metabolic fluxes according to cellular physiology.

Moreover, expanding the repertoire of microbial chassis beyond traditional chassis may offer new opportunities for producing specific classes of TCM compounds. Alternative chassis, including filamentous fungi, actinomycetes, photosynthetic microorganisms and plants, may provide more compatible metabolic and physiological environments for the biosynthesis of complex natural products, particularly those requiring specialized tailoring modifications or unique cofactors.

## 5 Conclusion and perspective

This special issue highlights recent advances in synthetic biology in the context of TCM-derived natural product research. The contributions to this field span a broad spectrum — from fundamental discoveries, such as the identification of novel biosynthetic enzymes and pathways in medicinal plants, to advanced metabolic engineering strategies aimed at optimizing production titers and hosts robustness. The combination of systems biology and synthetic biology is breaking down traditional barriers in the production of plant-derived pharmaceuticals, paving the way for more economical, sustainable, and scalable manufacturing paradigms. The continued integration of multi-approaches such as encompassing AI-driven pathway design, enzyme engineering, and next-generation bioprocessing will be essential to accelerate TCM bio-manufacturing.

